# Patients’ knowledge and beliefs concerning gout and its treatment: a population based study

**DOI:** 10.1186/1471-2474-13-180

**Published:** 2012-09-21

**Authors:** Leslie R Harrold, Kathleen M Mazor, Daniel Peterson, Nausheen Naz, Cassandra Firneno, Robert A Yood

**Affiliations:** 1 Meyers Primary Care Institute, Worcester, MA, USA; 2 University of Massachusetts Medical School, Worcester, MA, USA; 3 Reliant Medical Group, Worcester, MA, USA; 4 Fallon Community Health Plan, Worcester, MA, USA; 5 Department of Medicine, University of Massachusetts Medical School, Biotech 4, Suite 315, 377 Plantation Street, Worcester, MA 01605, USA

**Keywords:** Beliefs, Treatment, Gout, Dietary influence, Physician-patient communication

## Abstract

**Background:**

For patients to effectively manage gout, they need to be aware of the impact of diet, alcohol use, and medications on their condition. We sought to examine patients’ knowledge and beliefs concerning gout and its treatment in order to identify barriers to optimal patient self-management.

**Methods:**

We identified patients (≥18 years of age) cared for in the setting of a multispecialty group practice with documentation of at least one health care encounter associated with a gout diagnosis during the period 2008–2009 (n = 1346). Patients were sent a questionnaire assessing knowledge with regard to gout, beliefs about prescription medications used to treat gout, and trust in the physician. Administrative electronic health records were used to identify prescription drug use and health care utilization.

**Results:**

Two hundred and forty patients returned surveys out of the 500 contacted for participation. Most were male (80%), white (94%), and aged 65 and older (66%). Only 14 (6%) patients were treated by a rheumatologist. Only a minority of patients were aware of common foods known to trigger gout (e.g., seafood [23%], beef [22%], pork [7%], and beer [43%]). Of those receiving a urate-lowering medication, only 12% were aware of the short-term risks of worsening gout with initiation. These deficits were more common in those with active as compared to inactive gout.

**Conclusion:**

Knowledge deficits about dietary triggers and chronic medications were common, but worse in those with active gout. More attention is needed on patient education on gout and self-management training.

## Background

Gout is a common cause of inflammatory arthritis affecting up to 6 million Americans [1]. The prevalence of gout increases with advancing age and is associated with diuretic use, low dose aspirin, hypertension, cardiovascular disease, chronic renal insufficiency, and metabolic syndrome [2]. Both diet and alcohol intake have been shown to be risk factors for incident gout as well as triggers for recurrent attacks in those with the condition [3-5]. Specifically, higher levels of meat (beef and pork) and seafood consumption as well as beer and spirits (but not moderate amounts of wine) in gout patients are associated with gout flares [6,7]. Acute gouty symptoms are usually treated with nonsteroidal anti-inflammatory drugs (NSAIDs), colchicine, or glucocorticosteroids, while chronic gout is managed using urate-lowering therapy such as allopurinol, febuxostat, probenecid and sulfinpyrazone [8].

Self-management of gout by patients is complex due to the impact of diet and alcohol on symptoms as well as the different classes of medications used based on whether treatment is directed towards acute versus chronic symptoms [9]. Our preliminary work and that of others suggests that patients typically receive very little education on the dietary and lifestyle factors associated with gout [9,10]. These deficits in patient education may contribute to medication nonadherence. In particular, patients may be unaware of the risks of gout flares with initiation of urate-lowering medications [9]. Thus when flares occur, patients may assume the medication is not working and therefore stop taking it [9,11]. Additionally, they may be unaware or believe that medications are not to be used chronically since multiple studies have shown that the majority of patients do not use urate-lowering medications regularly [12-17]. In fact, a recent study comparing medication nonadherence across 7 common chronic conditions such as hypertension, osteoporosis, and diabetes mellitus found adherence was lowest with gout [18].

The purpose of this study was to identify patient knowledge and beliefs regarding gout and its management. We hypothesized that substantial numbers of patients are unaware of the impact of diet and alcohol intake on gout flares as well as the risks and benefits of chronic gout medications. A better understanding of these issues is critical in order to identify barriers to optimal patient self-management.

## Methods

We identified eligible patients based on enrollment in the Fallon Community Health Plan (FCHP) (a managed care plan) in Central and Eastern Massachusetts, United States. The patient study population included only members of the group-model component of the plan who received care from Reliant Medical Group (formerly Fallon Clinic), a multispecialty group practice. The computerized information system of the health plan contains records on utilization of all health care services including encounters and diagnostic testing as well as medical diagnoses and prescription drug dispensings. A total of 191,000 individuals were enrolled in the health plan in 2009. We identified members from the dataset who met criteria for enrollment into the cohort: age at least 18 years with at least one diagnosis code for gout (ICD-9 code 274.XX) in the prior 2 years (2008–2009) and were enrolled at least 12 months prior to the survey administration. There were 500 members contacted for participation in the study in the first 2 weeks of January 2010. The study was approved by institutional review boards at FCHP, Reliant Medical Group and the University of Massachusetts Medical School and patients provided consent prior to enrollment into the study.

### Health care utilization and status

Administrative health records were used to identify prescription drug dispensings, health care utilization, and inpatient and outpatient diagnosis in the 1 year prior to the survey administration. Diagnoses for gout and comorbidities were captured based on International Classification of Diseases 9^th^ Revision (ICD-9) codes used by physicians for administrative purposes. All patients in this study had prescription drug coverage through the managed care plan. Initiation of a urate-lowering drug was identified based on a dispensing of allopurinol and probenecid (no patients received sulfinpyrazone or febuxostat during the study period). Patients were asked to report using their gout-related health care encounters (ambulatory and emergency department) and procedures (injections and radiographic studies) in the questionnaire. Since self-reported health care utilization was similar to the administrative claims, we present here self-reported utilization (data not shown).

### Questionnaire content

The questionnaire was developed to include questions on the following topics: knowledge regarding gout (including etiology, dietary triggers, and the role of urate-lowering medications); beliefs about prescription medications used to treat gout (including ease of administration, effectiveness in decreasing pain, and associated side effects); overall health motivation; and trust in the physician. The items assessing overall health motivation and trust were drawn from existing measures [19-21]. The knowledge and belief items were developed by the authors for this present study. A number of background items were included (e.g., age, education, self-rated health). Preliminary versions of the questionnaire were pre-tested using cognitive interviews with a convenience sample of 5 patients in 1:1 sessions. A brief description of each set of items follows.

### Gout-related knowledge-*etiology and general principles of treatment*

The questions we developed to test knowledge of the etiology of gout were based on our previous in-depth interviews with patients in which we identified common deficits that impacted optimal self-management [9]. Two of the authors drafted the items (LRH, a rheumatologist and KMM, a psychometrician with experience in item writing). The draft items were reviewed and revised by all authors. Cognitive interviews were conducted using the draft items; the items were again reviewed and revised by the authors based on these results. The final set of questions included four items for patients to respond to with options of “True,” “False” and “Not Sure.” The statements were focused on the etiology of gout (e.g., “Gout is caused by too much uric acid,” “Gout flares can result from crystals forming in and around joints”), the need for chronic therapy as opposed to intermittent medication use in those started on urate-lowering therapy (e.g., “Medications which lower uric acid such as allopurinol or probenecid can be taken only when you have a gout flare”), and awareness of risks of flare triggered by medications (e.g., “Chronic gout medications such as allopurinol or probenecid can cause gout flares when the medications are started”). Each item was scored with 1 point given for each correct answer and 0 for incorrect or “Not Sure” responses.

### G**out related knowledge-*diet and alcohol consumption***

A 10-item test of dietary and alcohol products that can trigger gout was developed for this study as there were no previously developed instruments available in the published literature. The diet/alcohol test was developed based on available published literature that found an association between the consumption of these products and acute gouty symptoms and the current gout treatment recommendations which suggest providers should counsel patients on this topic [6,7,22,23]. Three response options included: “Yes, might cause a flare,” “No probably would not cause a flare,” and “Not sure.” Each item was scored with 1 point given for each correct answer and 0 for incorrect or “not sure” responses. A summary dietary knowledge score was created based on a total score (range 0–10 with the higher score reflecting greater knowledge).

### Beliefs about gout and medications

A total of 31 items were developed to assess beliefs about gout severity, gout medication efficacy, negative perceptions of prescription medications, and barriers to medication use. These items were developed based on the work by members of the research team (RAY and KMM) previously in osteoporosis and based on the health belief model [24,25]. These questions were revised to make them specific to gout by one author (LRH) and reviewed and edited by all authors. Draft items were tested in cognitive interviews, and all authors reviewed and revised the items before inclusion in the final questionnaire. Response options for all belief items were “Strongly Agree,” “Agree,” “Neutral,” “Disagree” and “Strongly Disagree.” The items were grouped into 3 scales: 1) beliefs in acute medications (6 questions each for nonsteroidal anti-inflammatory drugs, colchicine and glucocorticoids), 2) beliefs regarding the need for and effectiveness of chronic gout therapy as well as barriers to use of these chronic agents (9 questions), and 3) beliefs regarding prescription medications in general (3 questions).

### Health motivation

Lastly 4 questions were included on health motivation and adapted from the Osteoporosis Health Belief Scale [21,25]. These questions are not condition specific but rather assess overall motivation with regards to health. A sample question was “Keeping healthy is very important to me.” Response options were “Strongly Agree,” “Agree,” “Neutral,” “Disagree” and “Strongly Disagree” with higher scores suggesting greater health motivation. For this score, we combined the “Strongly agree” and “Agree” responses. Each item was scored with 1 point given for agreement to a statement suggesting strong health motivation and a 0 for “Neutral,” “Disagree,” and “Strongly Disagree” responses. The resulting range was 0–4 with the higher score reflecting greater health motivation.

#### Trust in the physician treating the gout

The short form of the trust scale was developed by Dugan and colleagues and consists of five questions [19]. An example from this set was “All in all, I have complete trust in my doctors.” Five response options were provided and were as follows: “Strongly disagree,” “Disagree,” “Uncertain,” “Agree” and “Strongly agree.” Responses were scored so that a higher score indicated higher trust. For example, for positive statements such as “I completely trust my doctor’s decisions about which medical treatments are best for me,” we assigned a score of 1 for a response of “Strongly agree” or “Agree” and a 0 for “Uncertain,” “Disagree,” and “Strongly Disagree.” For negative statements such “Sometimes my doctor cares more about what is convenient for him/her than my medical need,” we assigned a score of 1 for a response of “Strongly disagree” or “Disagree” and a 0 for “Uncertain,” “Agree,” and “Strongly Agree.” A summary trust in the physician score was created through taking the mean score across the five items (range 0–5).

#### Questionnaire administration

A random sample of 500 patients who met eligibility criteria were sent a letter informing them about the study. Patients were notified that they could opt out of the study by contacting a toll-free number. One week after the letter, those patients who did not decline to participate were sent a self-administered questionnaire with $5 cash and a research authorization and consent form permitting review of electronic medical records. Two reminders were sent to those not returning the questionnaire (each 10 to 14 days after the prior mailing).

### Analyses

#### Statistical analyses

Overall there was a low rate of missing data (<4%) for the specific instruments. We excluded missing values from the analyses. Cronbach’s alpha was computed to assess score reliability for gout knowledge on dietary triggers, general beliefs about prescription medications, health motivation, and trust in physician. Initial analyses were performed using descriptive statistics. For continuous variables we calculated means and standard deviations. For mean scores we calculated 95% confidence intervals. For categorical variable, proportions were calculated. We used Chi square or Fisher’s exact test for discrete variables and *t*-test or the Wilcoxon test for continuous variables. Specifically we identified patients with “active gout” which we defined as those patients reporting 2 or more gout flares that required medical care (an outpatient or Emergency Department visit) in the past 6 months and compared them to those without active gout. As sensitivity analyses, we also examined whether there were differences based on gout utilization (comparing those with ≥ 1 encounter of gout in the prior 12 months vs. those with none) and disease stage comparing those with receipt of a urate-lowering drug (used as a proxy for chronic gout) to those not on those agents.

## Results

Of the 500 eligible patients contacted for participation in the study including filling out the questionnaire and providing access to their claims data, 121 declined participation and 139 did not respond. This resulted in 240 patients who responded (response rate of 51% after excluding patients who reported never having gout n = 33; gout was likely a “rule out” diagnosis by providers, and upon review of additional evidence the patients were considered to not have gout). There were no significant differences between responders and nonresponders in terms of age, gender, and number of claims for gout. However, nonresponders were less likely to be dispensed a urate lowering drug (36% vs. 46%, p = 0.03). The baseline characteristics of the overall patient population are shown in Table 1. The majority of patients were male (80%), white (94%), and aged 65 and older (67%). Alcohol use, defined as having 2 or more drinks in the past 7 days, was reported by 45%. Comorbid conditions such as dyslipidemia (83%), hypertension (78%), diabetes (39%), and coronary heart disease (32%) were common. Based on pharmacy dispensings in the prior 12 months, 47% received a urate-lowering drug, 36% an NSAID, 26% colchicine, and 19% a glucocorticoid. Only 14 patients (6%) were cared for by a rheumatologist, and they were not more knowledgeable about dietary triggers; however, they were more likely to be aware of the risk of acute gouty symptoms with initiation of urate-lowering therapy (p =0.04).

**Table 1:** Characteristics of the gout patients who responded to the questionnaire

**Demographics**	**Total N = 240**	**Active gout N = 53**	**Inactive gout N = 187**	**P value**
Age (N and%)				0.016
<65	81 (34)	12 (23)	69 (37)	
65-74	93 (39)	17 (32)	76 (41)	
75 or older	66 (28)	24 (45)	42 (22)	
Gender (% male)	193 (80)	37 (70)	156 (83)	0.028
Race (N and%)				0.825
Caucasian	225 (94)	50 (94)	175 (94)	
Native American	9 (4)	2 (4)	7 (4)	
Hispanic	3 (1)	1 (2)	2 (1)	
Black	2 (1)	0 (0)	2 (1)	
Asian	1 (0)	0 (0)	1 (1)	
Highest grade completed (N and%)				0.331
No high school degree	33 (14)	9 (18)	24 (13)	
High school graduate	86 (36)	19 (37)	67 (36)	
College	118 (50)	23 (45)	95 (51)	
**Health Behaviors**				
Current smoking (N and%)	13 (5)	3 (6)	10 (5)	0.929
Alcohol use* (N and%)	94 (39)	13 (25)	81 (43)	0.014
**Associated Conditions**				
Dyslipidemia (N and%)	196 (82)	45 (85)	151 (82)	0.581
Hypertension (N and%)	185 (78)	44 (83)	141 (76)	0.295
Diabetes mellitus (N and%)	91 (38)	22 (42)	69 (37)	0.579
Coronary heart disease (N and%)	77 (32)	21 (40)	56 (30)	0.200
Renal disease (N and%)	43 (18)	12 (23)	31 (17)	0.327
Peripheral arterial disease (N and%)	21 (9)	8 (15)	13 (7)	0.069
Nephrolithiasis (N and%)	17 (7)	4 (8)	13 (7)	0.897
**Gout history**				
Gout encounters (mean, ± SD)**	1 (2)	2 (3)	1 (2)	
Cared for by a rheumatologist (N and%)	14 (6)	8 (19)	6 (4)	
ULD dispensing (N and%) **	108 (47)	37 (70)	77 (42)	0.0004
Colchicine use (N and%)**	59 (26)	23 (43)	37 (21)	0.0011
Glucocorticoid use (N and%)**	44 (19)	19 (36)	26 (14)	0.0005
NSAID use (N and%)**	72 (32)	14 (26)	60 (33)	0.355

* reports having 2 or more drinks of alcohol in the last 7 days.

** based on visits or dispensing of urate-lowering drugs (ULDs), colchicine, steroids and NSAIDs in the prior 12 months.

There were 53 patients (22%) who reported active gout (required ≥2 ambulatory or Emergency Department visits for active gout). They were more likely to be female (30% vs. 17%; p = 0.028), aged 75 and older (45% vs. 22%; p = 0.016) and less likely to report alcohol use (25% vs. 43%; p = 0.014) than those without active gout. They were also more likely to have received a prescription in the prior 12 months for a urate-lowering drug (70% vs. 41%; p = 0.0002), colchicine (43% vs. 21%; p = 0.002), and a glucocorticoid (36% vs. 14%; p = 0.001). While they were more likely to be cared for by a rheumatologist (18% vs. 4%; p =0.001), >80% were cared for solely by a primary care provider. In terms of gout-related self-reported health care utilization in the prior 6 months (Table 2), overall 44% reported ≥1 ambulatory encounters, 5% ≥1 emergency department visits, and 5% ≥1 joint injections with more utilization occurring in those with active gout. Additionally, those with active gout were more likely to report missing work in the last 6 months due to gout than those without active gout (13% vs. 4%; p = 0.001).

**Table 2:** Proportion of patients reporting health care utilization for gout over the prior months 6 months

**Type of Care**	**Total population**	**Stratified by disease activity**		
	**N (%)**	**Active gout N (%)**	**Not active gout N (%)**	**P value**
Ambulatory encounter	84 (35)	51 (96)	33 (18)	0.0001
Emergency Department encounter	12 (5)	12 (23)	0 (0)	0.0001
Joint injection	11 (5)	7 (13)	4 (2)	0.0008
Radiology study	33 (14)	20 (38)	13 (7)	0.0001

The vast majority of patients knew that gout was related to uric acid (89%) and flares were the result of crystals inducing inflammation in and around joints (80%); however only 25% of patients who received a urate-lowering medication in the past year were aware that these medications were to be used chronically and only 12% knew that initiation of urate-lowering drugs could worsen gout in the short term. Many participants were unaware of foods that may lead to a gout flare (mean score of 3.4 [±2.3]; range 0–10; alpha = 0.68) (Table 3). Specifically, more patients incorrectly reported vegetables (58%), chicken (55%) and legumes (39%) as triggers as compared to foods documented to cause gout flares including seafood (23%), beef (22%) and pork (7%). Only 43% knew that beer intake could increase the chances of a gout flare. Awareness of dietary triggers was worse in those with active gout (means score of 2.7 vs. 3.6; p value = 0.014).

**Table 3:** Patient knowledge of foods and alcoholic beverages and their risk of causing a gout flare

	**% agreement that item could cause a gout flare**			
**Food**	**Total population**	**Active gout**	**Not active gout**	**P value**
Published evidence of an association between gout episode and level of consumption* (% agreement that item could cause a gout flare)				
Shellfish	55	21	22	0.855
Beer	43	28	45	0.026
Hard liquor (whiskey, gin and vodka)	34	28	34	0.419
Seafood excluding shellfish	23	53	52	0.957
Beef	22	11	24	0.046
Pork	7	4	8	0.288
**No associations seen (% agreement that item could cause a gout flare)**				
Vegetables	58	47	59	0.131
Chicken	55	42	57	0.051
Legumes (beans and lentils)	39	32	39	0.356
Wine	13	4	15	0.030
**Overall score (**mean ± SD)	3.4 (2.3)	2.7 (2.2)	3.6 (2.2)	0.014

*Epidemiologic studies have identified a relationship between the dietary intake and gout flares.

For the management of acute flares, nonsteroidal anti-inflammatory drugs were considered by patients to be easy to take (86%) and effective in decreasing pain (78%) with only 23% having side effects to the agents. Among colchicine users, colchicine was considered easy to take (91%) and effective (76%) but 37% reported side effects to the medication. The responses did not differ based on disease activity. Views on the need for chronic gout therapy as well as barriers to the use of these agents were similar in those with and without active gout. However, those with active gout were less likely to report that chronic gout medications prevent gout flares (56.3% vs. 77.8%, p = 0.026). Both groups had similar responses when asked about beliefs in prescription medications overall including distrust or concerns (12.2 and 11.9 with a range of 1–20; alpha = 0.79). The majority of patients reported high levels of trust in their physician (mean score of 21.5 [±4.1]; range 5–25; alpha = 0.89) and high levels of health motivation (mean score of 23.0 [±4.1]; range 6–30; alpha = 0.80). The responses were not different based on gout activity.

## Discussion

Our study provides new information on patient knowledge and beliefs regarding gout management. Several deficits in knowledge were identified in the total population including dietary triggers, dosing of urate-lowering medications during flares, and the risk of this drug class in causing worsening gout in the short-term. However, the deficits were greater in those with active gout. In contrast, mean scores for health motivation, beliefs regarding the utility of prescription medications, and trust in the treating provider were high in all gout patients. The patients with acute gout were a unique subset with a greater predominance of women, lower reported use of alcohol and greater likelihood of being cared for by a rheumatologist. Limited conclusions can be made since we know very little in terms of burden of disease, prior treatments and duration of gout therapy as well as potential limitations to the use of urate-lowering therapy in this subpopulation. However, further investigation is needed into this subgroup so that health care providers can target them for intensive treatment in order to control gout.

A few epidemiologic studies have examined the relationship between dietary factors and uric acid levels and gout flares [4,6,7,26]. Specifically, there is an increased risk of gout with higher levels of beef, pork and seafood consumption [6]. Moderate consumption of purine-rich vegetables have not been associated with an increased risk of gout. However, over half the patients in our study believed that intake of vegetables was associated with a risk of flare while only about one quarter were aware of the true association with beef or seafood. Similarly, beer is well known to trigger recurrent gout attacks, often occurring within 24 hours after alcohol consumption [7]. However, in our study fewer than half of the patients reported beer as a potential cause of gout flares.

In this study, lack of awareness regarding the need for chronic therapy with urate-lowering medications as well as short-term risk of acute gout symptoms with initiation of this class of medications may help explain the results of larger epidemiologic studies, specifically that most patients who initiate a urate-lowering medication have large gaps in drug use over the first year [14-17] with most returning to treatment over the next 4 years [27]. While interruptions in medication drug use commonly occur due to financial concerns or side effects, it is less likely these factors played a role given the availability of generic medications with modest copayments and that most patients eventually restarted their prior urate-lowering medication.

Several factors may contribute to the patient knowledge deficits and incorrect medication beliefs identified here. In part, our findings may reflect how medical care is delivered in the US with a greater focus on acute illness rather than chronic disease management. Within the constraints of outpatient clinical encounters, it is challenging for providers to find time to teach patients self-management skills. Additionally, providers also may have very limited resources for patient education such as pamphlets on gout, ready access to reputable online patient-oriented education, and support staff for teaching and reinforcing concepts with patients. Furthermore, most providers have had little or no training in motivational interviewing or patient self-management training. We found that knowledge and medical beliefs were not higher in patients with greater health care utilization or chronicity (defined as receipt of a urate-lowering medication). This suggests that just recommending more visits with a provider will not necessarily result in better care. Additionally primary care providers may need more training in the pharmacologic and nonpharmacologic management of gout. Innovative approaches to improve care will need to engage both the provider and the patient.

Our study is similar to others in the United Kingdom identifying identify patient beliefs and knowledge deficits regarding dietary factors and medication usage in the management of gout [10,28]. However, the results presented here should be interpreted within the context of some limitations. The study is limited by the fact that this was based in a small managed care plan. However, we believe the sample represents a typical clinical practice with very few patients cared for by a rheumatologist and approximately half of the patients prescribed a urate-lowering drug. There was limited racial diversity in our population. In addition, we did not directly assess the conversations between provider and patients—only what the patient reported. Accordingly, we do not know the degree to which patient education was actually provided. Of note we did not assess health literacy which may have played a role with patients not being able to understand the instructions from the provider treating their gout [29].

## Conclusion

In this sample of gout patients cared mostly by primary care providers, there was limited knowledge regarding dietary triggers for gout suggesting better education is needed. There was also lack of awareness regarding the risk of gout flare with initiation of a urate-lowering medication. This suggests changes in current clinical practice should be considered. These include providing information regarding the short-term and long-term effects of urate-lowering medications and education on the impact of dietary factors on triggering an attack. These educational approaches should be presented in multiple modes (e.g., orally and in print) as well at multiple time points. Additionally these messages need to be reinforced in follow-up encounters or telephone calls.

## Competing interests

The authors declare that they have no competing interests.

## Authors’ contributions

LH conceived of the study, and was involved in the study design, acquisition of data, supervised the study analyses, reviewed the results, and provided critical input on the manuscript. KM participated in study design, review of results and drafting of the manuscript. DP was involved in acquisition of data, performed the study analyses and provided critical input on the manuscript. NN was involved in performing study analyses and drafting the manuscript. CF participated in acquisition of data, supervised the study analyses, and provided critical input on the manuscript. RY participated in study design, review of results and drafting of the manuscript. All authors read and approved the final manuscript.

## Pre-publication history

The pre-publication history for this paper can be accessed here:

http://www.biomedcentral.com/1471-2474/13/180/prepub
